# Implementation of an integrated care pathway for severe aortic stenosis: a prospective cohort study of clinical profiles, safety, and outcomes

**DOI:** 10.3389/fcvm.2025.1745204

**Published:** 2026-01-16

**Authors:** Violeta González-Salvado, Manuela Sestayo Fernández, Marta Alonso-Vázquez, Pablo de la Fuente-López, Joana Maria Laranjeira Correia, Uxía Lens-Bravo, Ánxel Ruzo-Cedillo, Bibiana Villamayor-Blanco, José Ramón González-Juanatey, Carlos Peña-Gil

**Affiliations:** 1 Cardiology Department, University Clinical Hospital of Santiago de Compostela, Santiago de Compostela, Spain; 2 Health Research Institute of Santiago de Compostela (IDIS), Santiago de Compostela, Spain; 3 Biomedical Research Networking Center on Cardiovascular Diseases (CIBERCV), Instituto de Salud Carlos III (ISCIII), Madrid, Spain; 4 Serviço de Cardiologia, Unidade Local de Saúde Tâmega e Sousa, Guilhufe, Portugal; 5 Rehabilitation Department, University Clinical Hospital of Santiago de Compostela, Santiago de Compostela, Spain

**Keywords:** aortic stenosis, aortic valve replacement, cardiac rehabilitation, health information management, integrated health care systems, postoperative complications, transcatheter aortic valve replacement

## Abstract

**Introduction:**

Severe aortic stenosis (AS) management increasingly requires multidisciplinary coordination across diagnostic, interventional, and rehabilitation stages. Integrated care pathways (ICPs) supported by structured data systems may improve safety and outcomes, but real-world evidence in AS remains limited. We aimed to characterize a contemporary cohort of patients with severe AS managed within an ICP at a tertiary hospital, comparing profiles and outcomes by treatment strategy, and to describe diagnostic procedures and the implementation of cardiac rehabilitation (CR) interventions embedded in the pathway.

**Methods:**

Prospective observational study of all consecutive patients with AS evaluated by a multidisciplinary heart team between 2018 and 2022. Baseline characteristics and frailty, diagnostic tests, CR interventions, procedural details, and outcomes were collected via an interoperable data management platform and compared across treatment groups: surgical (SAVR) or transcatheter (TAVR) valve replacement, or conservative management. Early survival was assessed using Kaplan–Meier analysis with log-rank testing.

**Results:**

Among 984 patients (median age 78 years, 42% women), 43.9% underwent SAVR, 49.8% TAVR, and 6.3% were managed conservatively. TAVR and conservative groups were older, frailer, and had higher comorbidity. Device success at 30 days was high (≈91%), with periprocedural death at 2.5% for interventions, vs. 9.7% early mortality for conservative management (*p* < 0.001). Early survival differed significantly (log-rank *p* = 0.004). TAVR had higher permanent pacemaker implantation (21.7% vs. 7.4% for SAVR, *p* < 0.001) and major vascular complications (4.1% vs. 0.2%, *p* < 0.001), while SAVR had more reoperations (8.3% vs. 0.2%, *p* < 0.001) and atrial fibrillation (18.9% vs. 10.1%, *p* = 0.001). Prehabilitation was implemented in 64.6% of candidates, while postprocedural CR remained underutilized (11.3%).

**Conclusions:**

Integrated care for a cohort of patients with AS, supported by structured data management, enabled comprehensive profiling, systematic outcome monitoring, and identification of improvement areas. Both SAVR and TAVR achieved high success with low early mortality, while conservative management had poor survival.

## 1 Introduction

Degenerative aortic valve stenosis (AS) is the third most common cause of heart disease and the valvular pathology with the greatest clinical and economic impact on healthcare systems across middle and high-income countries (1, 2). Its strong association with aging, contributes to a prevalence exceeding 12% in patients older than 75 years, with severe AS accounting for more than 3% of cases (3). Although the incidence of severe AS has remained relatively stable, the overall prevalence of calcific AS is expected to increase in the coming decades (4). Mortality from AS is strongly associated with symptom onset and has a poor prognosis if left untreated, with reported mortality rates around 45%–50% at five years (4, 5). Surgical (SAVR) or transcatheter (TAVR) aortic valve replacement remains the only therapeutic intervention shown to improve survival, and reduce morbidity in patients with symptomatic severe AS, and is indicated upon symptom onset or evidence of significant cardiac impairment (6, 7).

Traditional care models for AS have focused primarily on the valve replacement procedure. However, these models often rely on fragmented care delivered by multiple providers at different stages and settings, leading to a high risk of discoordination and care discontinuity that can limit their effectiveness. In contrast, integrated care pathways (ICPs) are structured, multidisciplinary care plans designed to maximize the effectiveness of clinical actions by enhancing coordination and continuity of care, with a patient-centered approach. Developing an ICP requires mapping each step of the patient's journey, clarifying professional roles, aligning interventions to maximize clinical value, and incorporating quality indicators for continuous evaluation and system improvement (8). Additionally, high-quality ICPs require tracking patients throughout the care pathway and linking care processes to individual health outcomes. ICPs with structured, interoperable data management enable standardized data collection and interpretation (9), facilitating reliable outcome analysis, quality monitoring, and comparisons over time and across populations and healthcare settings (10).

In the context of severe AS, structured data-supported ICPs offer particular advantages. First, disease presentation varies widely—from younger, fit individuals to frail elderly patients with multiple comorbidities—making standardized yet flexible care essential. Second, management typically involves multiple professionals across several stages, from diagnosis to intervention and rehabilitation, which benefits from coordinated, structured care (8). Third, there is a high risk of clinical deterioration and adverse events while awaiting valve intervention (11, 12). Continuous monitoring within an ICP may help ensure timely detection and response to clinical alerts, reducing the likelihood of missed adverse events, or gaps in follow-up. Finally, the large volume and complexity of clinical data generated throughout the pathway require interoperable systems that ensure consistent capture, integration, and effective application in guiding care.

The primary objective of this study was to analyze the clinical profile and clinical outcomes of a contemporary 5-year cohort of patients with severe AS managed within a defined AS-ICP supported by structured data management at a tertiary university hospital. We aimed to evaluate differences in patient baseline characteristics based on the type of treatment received—SAVR, TAVR, or conservative management—and to report short-term outcomes, including procedural success, complications, and early mortality. A secondary objective was to describe the diagnostic assessments and the implementation of prehabilitation and cardiac rehabilitation (CR) interventions embedded within the AS-ICP.

## 2 Methods

### 2.1 Study design, population and setting

We conducted a prospective observational study at a tertiary university hospital in Spain from January 2018 to December 2022, with clinical follow-up until November 2025. All consecutive adult (≥18 years) patients with severe AS evaluated by a multidisciplinary Heart Team and considered potential candidates for valve intervention were included. Patients with AS but whose primary diagnosis was active infective endocarditis were excluded to reduce heterogeneity.

The study complied with the Declaration of Helsinki and was approved by the Institutional Research Ethics Committee (Code 2020/405). Written informed consent was obtained from all participants.

### 2.2 Integrated care pathway for aortic stenosis (AS-ICP)

**Figure 1:**
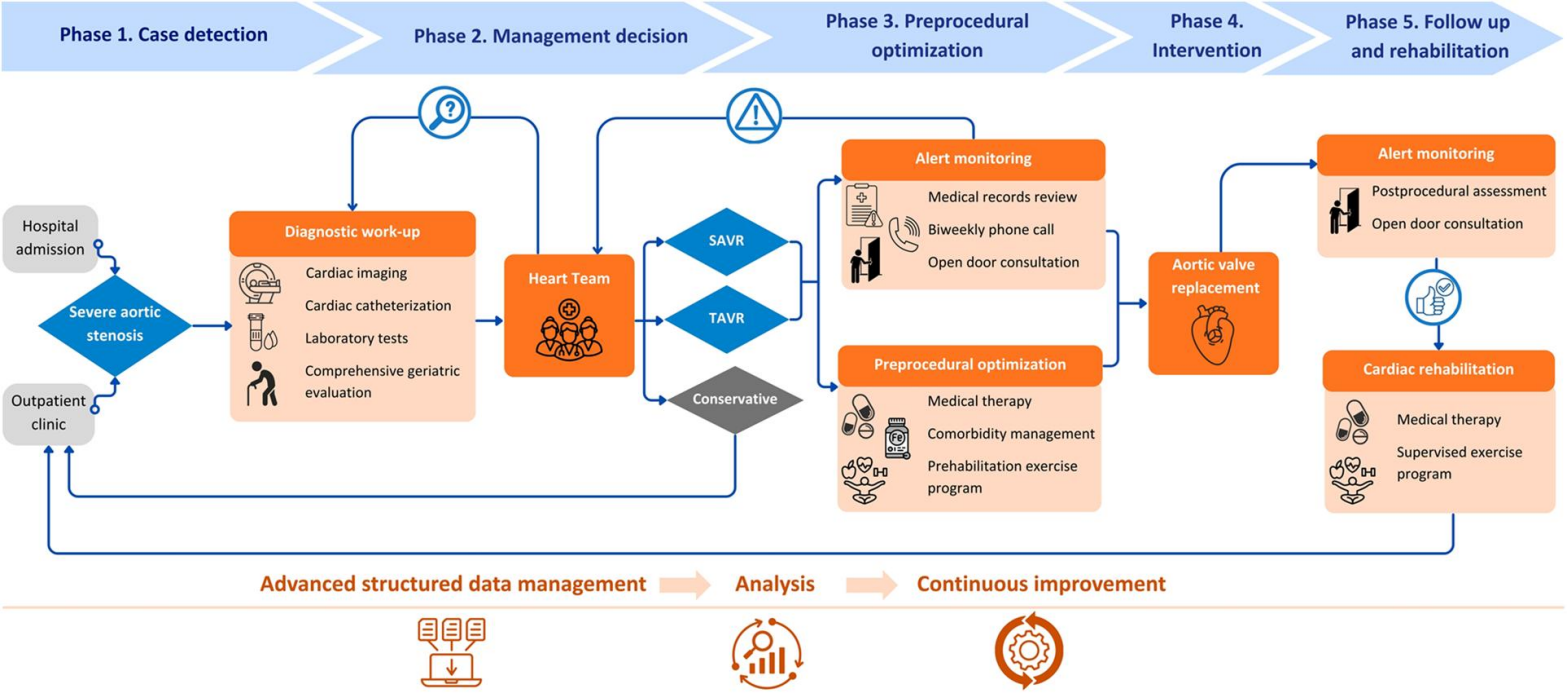
Aortic stenosis integrated care pathway (AS-ICP) flowchart with key interventions. SAVR, surgical aortic valve replacement; TAVR, transcatheter aortic valve replacement. Icons from Canva, licensed under Pro Content License.

The AS-ICP standardizes all diagnostic and therapeutic interventions for patients with severe AS who are candidates for valve replacement, from diagnosis to treatment and rehabilitation (Figure 1). At our center, it is led by two specialized case-management nurses and coordinated by two cardiologists. Since its implementation in 2018, the AS-ICP has evolved as a dynamic process adapted to the changing needs and conditions of the healthcare system.

Patients enter the AS-ICP following case review and discussion by the multidisciplinary Heart Team, including clinical and interventional cardiologists, cardiac rehabilitation and cardiac imaging specialists, cardiac surgeons, and specialized case-management nurses. Meetings are held twice weekly, and management decisions are reached by consensus and recorded electronically. These may include: SAVR, TAVR, conservative management, or further diagnostic evaluation with reassessment in a subsequent Heart Team meeting. Patients recommended for conservative management are referred for standard outpatient follow-up and formally exit the AS-ICP. Patients undergoing intervention remain under the care of the AS-ICP team. This includes patient education, adherence to diagnostic and therapeutic protocols (including geriatric assessment where indicated), clinical optimization before intervention (prehabilitation), and proactive monitoring and management. Monitoring is achieved via biweekly telephone follow-up by specialized case-management nurses, regular review of medical records, and an “open-door consultation” policy providing patients with direct access to the team in case of symptom progression or adverse events.

Prehabilitation prior to valve replacement includes pharmacological optimization and comorbidity management, including weight management in patients with obesity (with a body mass index of ≥35 kg/m^2^ as the referral threshold for endocrinology), and evaluation and treatment of anemia. Only patients without prior specific management before Heart Team presentation were counted as new referrals for these interventions. The program also comprises comprehensive physical and functional assessment by a rehabilitation physician, and prescription of an individualized exercise program including respiratory muscle training, supervised by a physical therapist. Following intervention, and in the absence of complications, patients are offered participation in an outpatient exercise-based CR program, typically consisting of 8–10 sessions twice a week. Upon completion, patients transition to routine cardiology follow-up and formally exit the AS-ICP. Importantly, during the COVID-19 pandemic in 2020, in-person prehabilitation and CR exercise sessions were suspended. Instead, we provided patients with home-based training materials, including instructional guides and videos created by the team.

### 2.3 Variables definition and data management

At inclusion, demographic, anthropometric, and clinical data—including comorbidities, symptoms, frailty, and physical performance—were prospectively collected. Laboratory and cardiac imaging parameters at Heart Team assessment were also recorded. AS phenotypes [high-gradient, low-gradient/low-flow with reduced left ventricular ejection fraction (LVEF), paradoxical low-gradient/low-flow with preserved LVF, and low-gradient/normal-flow] were defined according to current recommendations (13). The extent of AS-related cardiac damage was classified using the five-stage framework proposed by Généreux et al. (14). Patients aged ≥75 years—or younger if frailty was suspected—underwent a standardized multiparametric geriatric assessment conducted by the AS-ICP case manager nurses, provided the patient was in suitable condition for reliable testing. This assessment included evaluation of functional independence, social support, cognition, nutrition, frailty, and physical performance. Specific tools and interpretation details are available in the Supplementary Material.

All diagnostic tests and interventions aimed at clinical optimization were recorded. Procedural outcomes for patients undergoing aortic valve replacement (SAVR or TAVR)—including success rates, complications, and mortality—were recorded following Valve Academic Research Consortium-3 (VARC-3) criteria (15) (see Supplementary Material). Periprocedural mortality was defined as all-cause death within 30 days, or later if the patient remained hospitalized after the intervention (censoring at 84 days, corresponding to maximum observed hospitalization in a deceased patient). In the conservative group, mortality was measured from the date of the last Heart Team meeting. For survival analyses, time to event was calculated from the intervention date (SAVR/TAVR) or Heart Team decision (conservative group) to death or censoring at 84 days.

All clinical data (more than 300 variables per patient) were collected using a terminology dictionary largely interoperable with Systematized Nomenclature of Medicine Clinical Terms (SNOMED CT) and structured according to a defined clinical taxonomy with associated contexts and attributes. Data entry was performed via the naevia medical© platform (DILEMMA SOLUTIONS SL), which supports advanced medical data management and rule-based clinical decision support (16, 17). The system incorporates natural language processing tools for semi-automated, supervised extraction of unstructured data from medical texts, semantic logic for defining dependencies and generating inferences, and automated calculation tools. Each data entry is timestamped, allowing longitudinal analyses of care processes and outcomes.

### 2.4 Statistical analysis

Continuous variables are expressed as medians with interquartile ranges (IQR, Q1–Q3), and categorical variables as absolute values and percentages. Group comparisons between treatment strategies (SAVR, TAVR, conservative) were performed using Chi^2^ or Fisher's exact test for categorical variables, and Kruskal–Wallis or Mann–Whitney *U*-tests for continuous variables, as appropriate. Early mortality was analyzed using Kaplan–Meier survival analysis and compared with the log-rank test. The time-to-event variable and definition of periprocedural mortality were applied as previously described. In addition, stratified analyses within the SAVR and TAVR groups were performed to explore the association between prehabilitation and periprocedural or 30-day mortality, using Fisher's exact test due to the low event rate. Multivariable adjustment was not performed to avoid overfitting.

Statistical significance was defined as a two-sided *p*-value <0.05. Analyses were performed independently by a professional statistical team (Biostatech SL) using R version 4.2.

## 3 Results

### 3.1 Patients inclusion and Heart Team decision

During the study period, all consecutive patients with AS evaluated by the Heart Team were screened for inclusion. Nine patients with active infective endocarditis were excluded *a priori* to reduce clinical heterogeneity. A total of 988 patients were included in the first five years of the AS-ICP. Of these, 539 (54.6%) were presented to the Heart Team from the outpatient clinic and 449 (45.4%) during hospitalization. The mean annual inclusion rate was 198 ± 28 patients, increasing from 147 in 2018 (the first year of AS-ICP implementation) to >200 patients/year thereafter, except for a slight decline in 2020, likely related to the COVID-19 pandemic (216 in 2019, 187 in 2020, 225 in 2021, and 213 in 2022).

At the first Heart Team meeting, aortic valve replacement was recommended in 88% of cases (43% SAVR, 45% TAVR). Other decisions included the need for additional diagnostic testing (6.4%), conservative management despite a formal indication for intervention (3.8%), and contraindication to intervention (1.5%). A total of 146 patients (14.8%) were re-evaluated in a subsequent Heart Team meeting. The initial decision was upheld in most patients (*n* = 903, 91.4%), while therapeutic approach was modified in 85 patients (8.6%), as detailed in Supplementary Figure S1. These changes were mainly due to significant clinical updates, but also to patient refusal of the initially proposed intervention (*n* = 32, 3.2%). Among patients managed conservatively, the decision was primarily driven by clinical contraindications identified by the Heart Team, including advanced comorbidity or frailty leading to procedural futility and/or unfavorable anatomy (*n* = 51). In addition, 9 patients declined intervention, and in 2 cases no formal indication for valve replacement was longer found after reassessment. Four patients (0.4%) died before intervention (two patients assigned to SAVR, one to TAVR, and one patient pending additional tests for decision) and were excluded from the final analysis.

### 3.2 Patient characteristics by treatment group

#### 3.2.1 Comorbidity and clinical profile

We analyzed data from 984 patients: 432 (43.9%) underwent SAVR, 490 (49.8%) received TAVR, and 62 (6.3%) were managed conservatively (Table 1). Median age was 78 years (IQR 11), with two-thirds ≥75 years; 42.2% were women. Patients in the TAVR and conservative groups were older and more frequently female. Cardiovascular risk factors were highly prevalent, particularly overweight and grade I obesity, hypertension, and dyslipidemia. Cardiac comorbidities were similar across groups, except for a higher prevalence of atrial fibrillation in the TAVR and conservative groups. Extracardiac comorbidities were also more frequent in these groups.

**Table 1:** Baseline characteristics by treatment strategy.

Variables	Total (*n* = 984)	SAVR (*n* = 432)	TAVR (*n* = 490)	Conservative (*n* = 62)	*p*-value
Median age (years)	78 (11)	73 (10)	82 (8)	83 (10.75)	**<0** **.** **001**
Age group					
<65 years	76 (7.7%)	67 (15.5%)	5 (1.0%)	4 (6.5%)	**<0.001**
65–74 years	257 (26.1%)	202 (46.8%)	47 (9.6%)	8 (12.9%)	
75–84 years	463 (47.1%)	161 (37.3%)	275 (56.1%)	27 (43.5%)	
≥85 years	188 (19.1%)	2 (0.5%)	163 (33.3%)	23 (37.1%)	
Female sex	415 (42.2%)	149 (34.5%)	239 (49.8%)	27 (43.6%)	**<0** **.** **001**
Anthropometrics					
BMI (kg/m^2^)	29.0 (6.1)	29.2 (5.5)	28.9 (6.3)	27.7 (6.9)	0.224
BMI category					
Underweight	2 (0.2%)	2 (0.5%)	–	–	0.432
Normal range	187 (19.0%)	71 (16.4%)	99 (20.2%)	17 (27.4%)	
Overweight	393 (39.9%)	174 (40.3%)	195 (39.8%)	24 (38.7%)	
Obesity	402 (40.9%)	185 (42.8%)	196 (33.9%)	21 (33.9%)	
Grade of obesity					
Grade 1	298 (30.3%)	150 (34.7%)	132 (26.9%)	16 (25.8%)	0.051
Grade 2	75 (7.6%)	27 (6.3%)	44 (9.0%)	4 (6.5%)	
Grade 3	29 (3.0%)	8 (1.9%)	20 (4.1%)	1 (1.6%)	
Cardiac and extracardiac comorbidity					
Hypertension	791 (80.4%)	326 (75.5%)	412 (84.1%)	53 (85.5%)	**0** **.** **002**
Dyslipidemia	699 (71.0%)	327 (75.7%)	336 (68.6%)	36 (58.1%)	**0** **.** **004**
Diabetes mellitus	350 (35.6%)	162 (37.5%)	164 (33.5%)	24 (38.7%)	0.384
Smoker	44 (4.5%)	26 (6.0%)	14 (2.9%)	4 (6.5%)	0.050
Atrial fibrillation	287 (29.2%)	94 (21.8%)	163 (33.3%)	30 (48.4%)	**<0** **.** **001**
Coronary artery disease	277 (28.2%)	107 (24.8%)	155 (31.6%)	15 (24.2%)	0.053
Myocardial infarction	113 (11.5%)	42 (9.7%)	64 (13.1%)	7 (11.3%)	0.284
Peripheric artery disease	87 (8.8%)	28 (6.5%)	52 (10.6%)	7 (11.3%)	0.069
Ischemic stroke or TIA	53 (5.4%)	20 (4.6%)	25 (5.1%)	8 (12.9%)	**0** **.** **024**
Hemorrhagic stroke	12 (1.2%)	2 (0.5%)	9 (1.8%)	1 (1.6%)	0.159
Gastrointestinal bleeding	76 (7.7%)	26 (6.0%)	41 (8.4%)	9 (14.5%)	**0** **.** **048**
Cancer	147 (14.9%)	45 (10.4%)	88 (18.0%)	14 (14.5%)	**0** **.** **001**
Chronic kidney disease	169 (17.2%)	46 (10.7%)	101 (20.6%)	22 (35.5%)	**<0** **.** **001**
COPD	96 (9.8%)	30 (6.9%)	61 (12.5%)	5 (8.1%)	**0** **.** **017**
Active interventions or treatments					
Dialysis	12 (1.2%)	5 (1.2%)	5 (1.0%)	2 (3.2%)	0.325
Previous cardiac surgery^a^	25 (2.5%)	4 (0.9%)	18 (3.7%)	3 (4.8%)	**0** **.** **015**
CIED	63 (6.4%)	15 (3.5%)	44 (9.0%)	4 (6.5%)	**0** **.** **003**
Oral anticoagulation	274 (27.9%)	93 (21.5%)	155 (31.6%)	26 (41.9%)	**<0** **.** **001**
Surgical risk scores^b^					
Euroscore II (%)	2.4 (2.2)	1.6 (1.7)	3.4 (2.4)	3.7 (7.6)	**<0** **.** **001**
STS mortality (%)	2.4 (2.2)	1.7 (1.2)	3.4 (2.4)	4.3 (3.8)	**<0** **.** **001**
STS morbimortality (%)	13.3 (9.2)	11 (6.5%)	16.8 (10)	18.1 (14.3)	**<0** **.** **001**
Clinical presentation					
Asymptomatic	19 (1.9%)	11 (2.6%)	6 (1.2%)	2 (3.2%)	0.259
Dyspnea NYHA class					
I	72 (7.3%)	42 (9.7%)	22 (4.5%)	8 (12.9%)	**<0.001**
II	425 (43.2%)	225 (52.1%)	183 (37.4%)	17 (27.4%)	
III	387 (39.3%)	140 (32.4%)	215 (43.9%)	32 (51.6%)	
IV	100 (10.2%)	25 (5.8%)	70 (14.3%)	5 (8.1%)	
Angina CCS class					
No angina or class I	735 (74.7%)	300 (69.4%)	385 (78.6%)	50 (80.7%)	**0.018**
II	160 (16.3%)	91 (21.1%)	62 (12.7%)	7 (11.3%)	
III	79 (8.0%)	38 (8.8%)	37 (7.6%)	4 (6.5%)	
IV	10 (1.0%)	3 (0.7%)	6 (1.2%)	1 (1.6%)	
Syncope	99 (10.1%)	57 (13.2%)	36 (7.4%)	6 (9.7%)	**0** **.** **013**
Laboratory parameters					
Hemoglobin (g/dL)	13.3 (2.4)	13.8 (2.2)	12.8 (2.2)	12.5 (2.4)	**<0** **.** **001**
Seric albumin (g/dL)	4.2 (0.5)	4.3 (0.5)	4.1 (0.5)	4.0 (0.7)	**<0** **.** **001**
NT-proBNP (pg/mL)	1,341 (3,028)	832 (1,357)	1,906 (3,770)	2,784 (5,943)	**<0** **.** **001**
CKD-EPI GFR (mL/min/1.73 m^2^)	70.6 (34.3)	81.7 (27.6)	61.7 (32.3)	52.8 (41.4)	**<0** **.** **001**
GFR category					
G1	188 (19.1%)	141 (32.6%)	41 (8.4%)	6 (9.7%)	**<0.001**
G2	441 (44.8%)	205 (47.5%)	217 (44.3%)	19 (30.6%)	
G3A	201 (20.4%)	60 (13.9%)	122 (24.9%)	19 (30.6%)	
G3B	99 (10.1%)	14 (3.2%)	72 (14.7%)	13 (21.0%)	
G4	40 (4.1%)	5 (1.2%)	33 (6.7%)	2 (3.2%)	
G5	15 (1.5%)	7 (1.6%)	5 (1.0%)	3 (4.8%)	

BMI, body mass index; TIA, transient ischemic attack; COPD, chronic obstructive pulmonary disease; CIED, cardiac implantable electronic devices; NYHA, New York Heart Association; CCS, Canadian Cardiovascular Society; NT-proBNP, N-terminal pro B-type natriuretic peptide; GFR, glomerular filtration. Numeric variables are expressed as median values (IQR).

Bold values indicate a significantly statistical difference.

^a^ Prior surgical aortic valve replacement was present in 6 TAVR patients, in 1 patient in the conservative group, and in no patients in the SAVR group.

^b^ Scores were available for 573 patients using EuroSCORE II (291 SAVR, 252 TAVR, 30 conservative) and for 441 using STS (217 SAVR, 206 TAVR, 18 conservative).

Dyspnea was the predominant symptom, especially *New York Heart Association* (NYHA) classes II and III, with a significant tendency to higher classes in the TAVR and conservative groups. Moderate to severe angina (*Canadian Society of Cardiology*, CSC classes II-III) was more frequently reported in the SAVR group, which also had a higher prevalence of syncope.

Laboratory parameters showed a graded pattern across treatment groups. Hemoglobin, albumin levels, and glomerular filtration rate were lowest in the conservative group, followed by TAVR and SAVR groups, while N-terminal pro B-type natriuretic peptide (NT-proBNP) levels showed the opposite trend.

#### 3.2.2 Aortic stenosis phenotype and related cardiac damage

Echocardiographic characteristics are presented in Table 2. Overall, structural and functional impairment was greatest in the conservative group. Consistently, cardiac damage as defined by Généreux et al. (14) progressed from SAVR to TAVR and was most advanced in the conservative group (Stages 3–4: ≈10% in SAVR, ≈21% in TAVR, ≈40% in conservative).

**Table 2:** Aortic stenosis phenotypes and cardiac damage.

Variables	Total (*n* = 984)	SAVR (*n* = 432)	TAVR (*n* = 490)	Conservative (*n* = 62)	*p*-value
LV ejection fraction (%)	61 (15)	62 (12)	61 (16)	55 (25)	**0** **.** **003**
LV indexed end-diastolic volume (mL/m^2^)	55 (23)	56 (21)	54 (25)	56 (26)	0.611
LV indexed end-systolic volume (mL/m^2^)	22 (17)	21 (15)	21 (18)	24 (24)	0.526
LV indexed mass (g/m^2^)	139.6 (53)	133.5 (50.3)	144.9 (52.6)	138.3 (49.4)	**<0** **.** **001**
LA indexed volume (mL/m^2^)	45 (20)	40 (16)	48 (22)	50 (23)	**<0** **.** **001**
RV basal diameter (mm)	37 (6)	36 (6)	37 (7)	38 (6)	0.136
RV dilation	123 (12.5%)	33 (7.6%)	79 (16.1%)	11 (17.7%)	**<0** **.** **001**
TAPSE (mm)	20 (5)	21 (4)	20 (5)	19 (4)	**<0** **.** **001**
Aortic stenosis characteristics					
Aortic peak gradient	80 (28)	81 (29)	78 (27)	77 (28)	**0** **.** **029**
Aortic mean gradient	48 (18)	50 (19)	48 (17)	45 (19)	**0** **.** **010**
Calcium score^a^	2,938 (1,735)	2,275 (1,201)	2,990 (1,702)	3,333 (3,018)	0.054
Bicuspid aortic valve	108 (11%)	88 (20.4%)	13 (2.7%)	7 (11.3%)	**<0** **.** **001**
Very severe AS	287 (29%)	137 (31.7%)	136 (27.8%)	14 (22.6%)	0.209
Rapidly progressive AS	240 (24.4%)	104 (24.1%)	124 (25.3%)	12 (19.4%)	0.577
Moderate to severe AS progression (months)^b^	25.6 (28.8)	24.4 (26.6)	25.6 (30.5)	28.1 (29.1)	0.419
AS phenotype					
High gradient AS	842 (85.6%)	381 (88.2%)	416 (84.9%)	45 (72.6%)	**<0.001**
LGLF AS (with reduced LVEF)	72 (7.3%)	19 (4.4%)	46 (9.4%)	7 (11.3%)	
Paradoxical LGLF AS (with preserved LVEF)	51 (5.2%)	21 (4.9%)	25 (5.1%)	5 (8.1%)	
Low gradient normal flow AS	19 (1.9%)	11 (2.6%)	3 (0.6%)	5 (8.1%)	
Cardiac damage staging (as defined by Généreux et al.)					
Stage 0	1 (0.1%)	1 (0.2%)	–	–	**<0.001**
Stage 1	214 (21.8%)	134 (31%)	74 (15.1%)	6 (9.7%)	
Stage 2	599 (60.9%)	255 (59%)	312 (63.7%)	32 (51.6%)	
Stage 3	127 (12.9%)	33 (7.6%)	82 (16.7%)	12 (19.4%)	
Stage 4	43 (4.4%)	9 (2.1%)	22 (4.5%)	12 (19.4%)	
Concomitant relevant valve disease and/or aortic dilatation					
Moderate to severe AR	150 (15.2%)	70 (16.2%)	71 (14.5%)	9 (14.5%)	0.589
Moderate to severe MR	198 (20.1%)	62 (14.4%)	114 (23.3%)	22 (35.5%)	**<0** **.** **001**
Moderate to severe MS	17 (1.7%)	10 (2.3%)	7 (1.4%)	–	**0** **.** **008**
Moderate to severe TR	137 (13.9%)	35 (8.1%)	84 (17.1%)	18 (29%)	**<0** **.** **001**
Aortic root aneurysm	35 (3.6%)	24 (5.6%)	9 (1.8%)	2 (3.2%)	**0** **.** **010**
Ascending aorta aneurysm	46 (4.7%)	35 (8.1%)	7 (1.4%)	4 (6.5%)	**<0** **.** **001**

LV, left ventricle; RV, right ventricle; LA, left atrium; TAPSE, tricuspid annular plane systolic excursion; AS, aortic stenosis; LGLF, Low gradient low flow; AR, aortic regurgitation; MS, mitral stenosis; TR, tricuspid regurgitation. Numeric variables expressed as median values (IQR).

Bold values indicate a significantly statistical difference.

^a^ Calcium score (by chest CT) was available for *n* = 214 patients (23 SAVR, 180 TAVR, 11 conservative).

^b^ Date of moderate AS first diagnosis was available for 413 patients (176 SAVR, 204 TAVR, 33 conservative).

Patients who underwent SAVR had higher transaortic gradients and a greater prevalence of bicuspid aortic valve. While high-gradient AS was the most prevalent phenotype overall, low-gradient/low-flow AS phenotypes were more common in the TAVR and conservative groups. Very severe AS (mean gradient ≥60 mmHg or peak velocity ≥5.0 m/s) (6, 7) was observed in 27% overall, declining from 29.9% in 2018 to 23.5% in 2022 (Spearman's rho = –0.062; *p* = 0.052). Additionally, almost 25% of patients showed rapid progression from moderate to severe AS (increase in peak aortic jet velocity of ≥0.3 m/s per year) (6, 7) with a median progression interval of 25.6 months (IQR 28.8). Significant aortic regurgitation or dysfunction of other valves was frequently observed. Moderate to severe mitral and tricuspid regurgitation were more common among TAVR and conservative groups, while aortic regurgitation was slightly more frequent in SAVR.

#### 3.2.3 Multidimensional geriatric assessment

Frailty assessment was performed in 560 patients (57% of the cohort, 81% of those over ≥75 years): 24% in the SAVR, 70% in TAVR, and 63% in conservative group, with significant differences across groups (Table 3). The conservative group consistently showed the worst performance across all domains, including lower independence, higher cognitive decline, greater frailty, and poorer physical performance. In contrast, SAVR patients displayed the most favorable profile, while TAVR patients fell in between.

**Table 3:** Multidimensional geriatric profile by treatment group.

Variables	Valid values^a^	Total (*N* = 560)	SAVR (*N* = 132)	TAVR (*N* = 389)	Conser- vative (*N* = 39)	*p*-value
Functional independence & social support						
Barthel index	*N* = 560	100 (90,100)	100 (96.3,100)	95 (90,100)	85 (75,100)	**<0** **.** **001**
Gijón modified scale	*N* = 434	5 (4,7)	4 (3,6)	5 (4,7)	6 (4,8)	**0** **.** **012**
Cognitive & nutritional status						
MNA-SF	*N* = 546	12 (11,14)	13 (12,14)	12 (11,14)	12 (11,13)	**0** **.** **004**
Mini-mental test (Lobo)	*N* = 547	28 (26,30)	29 (28, 30)	28 (25,29)	26 (21, 29)	**<0** **.** **001**
Frailty & physical performance						
Frail scale	*N* = 443	2 (3)	1 (0,2)	2 (1,3)	3 (2,4)	**<0** **.** **001**
Essential frailty tooltest	*N* = 530	1 (0,3)	0 (0,1)	1 (0,2)	2 (1,3)	**<0** **.** **001**
Gait speed (m/s)	*N* = 552	0.85 (0.67, 0.99)	1.00 (0.86, 1.12)	0.82 (0.65, 0.96)	0.72 (0.52, 0.87)	**<0** **.** **001**
SPBB	*N* = 540	9 (6, 11)	10 (9,12)	8 (6,10)	6 (3,9)	**<0** **.** **001**
6-min Walking Test (m)	*N* = 514	300 (212, 364)	363 (300, 408)	283 (204, 343)	231 (143, 311)	**<0** **.** **001**
6-min Walking Test (% predicted)		80 (59,97)	91 (75,102)	76 (59, 95)	62 (35, 87)	**<0** **.** **001**

MNA-SF, Mini-nutritional assessment Short Form; SPBB, Short Physical Performance Battery. Interpretation is provided in the Supplementary Material. Numeric variables expressed as median values (Q1, Q3).

Bold values indicate a significantly statistical difference.

^a^ Detailed of missing or non-interpretable values for each test: MNA: 9 TAVR, 5 conservative; Mini-mental test: 1 SAVR, 10 TAVR, 2 conservative; Frail scale: 36 SAVR, 74 TAVR, 7 conservative; Essential Frailty Tooltest: 7 SAVR, 20 TAVR, 3 conservative; Gait speed: 5 TAVR, 3 conservative; SPBB: 3 SAVR, 14 TAVR, 3 conservative; 6-min Walking test: 5 SAVR, 31 TAVR, 10 conservative; Gijon scale: 39 SAVR, 76 TAVR, 11 conservative.

Differences between SAVR and TAVR remained significant for all domains, confirming different frailty and functional profiles even within the interventional arms (*p* < 0.05). Comparisons between TAVR and conservative groups were also significant (*p* < 0.05 for all variables), except for social support (Gijón scale, *p* = 0.149) and nutrition (Mini-Nutritional Assessment-Short Form, *p* = 0.063).

### 3.3 Diagnostic work-up and periprocedural optimization within the AS-ICP

We present detailed diagnostic data by treatment group in Supplementary Table S1 and by year in Supplementary Figure S2. The diagnostic approach in the interventional arms varied notably by treatment pathway. TAVR patients more frequently underwent non-invasive imaging, particularly cardiac CT angiography (99.8% vs. 27.3% for SAVR and 54.8% for conservative management, *p* < 0.001) and pyrophosphate scintigraphy (18.6% vs. 2.3% vs. 4.8%, *p* < 0.001). In contrast, SAVR patients underwent transesophageal echocardiography (25% vs. 12.9% for TAVR and 19.4% for conservative, *p* < 0.001), non-contrast chest CT (23.8% vs. 14.1% vs. 16.1%, *p* < 0.001), and supra-aortic trunk Doppler ultrasound (10.4% vs. 3.7% vs. 3.2%, *p* < 0.001) more often. Patients managed conservatively underwent fewer invasive procedures, but showed the highest use of stress testing (14.5% vs. 12% for SAVR vs. 7.1% for TAVR, *p* < 0.001).

Among patients referred for aortic valve replacement, a supervised prehabilitation exercise program was implemented in 64.6%, with significantly higher uptake among SAVR compared with TAVR patients (77.8% vs. 53.1%, *p* < 0.001). Participation in post-intervention CR was overall low (11.3%) but remained significantly higher after SAVR than after TAVR (16% vs. 7.1%, *p* < 0.001). Participation in both prehabilitation and rehabilitation exercise programs fell sharply in 2020 due to COVID-19 restrictions, with reductions of 63% and 73%, respectively, compared with 2019. By 2022, participation had risen significantly, exceeding pre-pandemic rates (Supplementary Figure S3). Anemia workup and treatment was implemented within the AS-ICP in 13% of the total cohort and in 51% of patients with moderate anemia (hemoglobin <11 g/dL), more frequently in the TAVR group (16.5% vs. 9.7%, *p* = 0.002). Obesity consultation was initiated in ≈9% of patients overall (7.8% TAVR vs. 11.3% SAVR, *p* = 0.063), and in 54% of patients with grade ≥ II obesity. No distinct temporal trends were observed for the latter interventions.

### 3.4 Characteristics and outcomes of valve replacement procedures

Details of aortic valve replacement procedures are shown in Supplementary Figure S4. Although isolated replacement was the most common overall, nearly 50% of SAVR patients and 8% of those undergoing TAVR received combined procedures. In SAVR, coronary artery bypass grafting was performed in 25% of cases, followed by ascending aorta and mitral valve replacement. Bioprosthetic valves were used in 75.9% of cases. In TAVR, concomitant coronary angioplasty was the most frequent procedure (6%). Transfemoral access was used in 95% of cases, followed by axillary (3.9%) and other approaches (0.6%). Self-expanding valves were implanted in 96% of cases [Corevalve/Evolut (Medtronic) 71%, ACURATE (Boston Scientific) 23.5%, others 1.5%].

Median hospital stay for intervention was 11 days (IQR 7, 20), significantly shorter for TAVR compared to SAVR patients, and for ambulatory patients compared to those referred during hospitalization. Among ambulatory patients, median stay was 6 days (IQR 5.9) for TAVR and 10 days (IQR 8.13) for SAVR, while median stay increased to 17 days (IQR 9.25) for TAVR and 22 days (IQR 14.32) for SAVR among those referred during hospitalization (Supplementary Figure S5).

Procedural outcomes are summarized in Table 4. Technical and device success rates were very high for both surgical and transcatheter approaches. Permanent pacemaker implantation was the most common complication, particularly after TAVR, which also showed higher rates of major vascular complications. Conversely, surgical reintervention (primarily due to postoperative bleeding or tamponade, with only one valve-related intervention for infective endocarditis) and atrial fibrillation were more common after SAVR. During early follow-up, 29/984 patients died: 11 in the SAVR group (2.5%), 12 in the TAVR group (2.4%), and 6 in the conservative group (9.7%) (*p* < 0.001). Overall periprocedural mortality remained stable throughout the study period, except for a slight peak in 2021 (3.3%). In an exploratory stratified analysis, periprocedural or 30-day mortality was numerically lower among patients undergoing prehabilitation in both SAVR (3.0% vs. 1.9%, *p* = 0.562) and TAVR (6.3% vs. 1.5%, *p* = 0.018) cohorts. Given the low event rate, these findings should be interpreted with caution.

**Table 4:** Success and complications of aortic valve replacement procedures.

Variables	Total (*n* = 922)	SAVR (*n* = 432)	TAVR (*n* = 490)	*p*-value
Procedural success^a^				
Technical success (at exit from procedure)	916 (99.3%)	431 (99.8%)	485 (99.0%)	0.223
Device success (at 30 days)	840 (91.1%)	389 (90%)	452 (92.2%)	0.299
Periprocedural complications^a^				
Permanent pacemaker implantation^c^	128 (14.8%)	31 (7.4%)	97 (21.7%)	**<0** **.** **001**
New-onset atrial fibrillation^b^	97 (14.6%)	64 (18.9%)	33 (10.1%)	**0** **.** **001**
Major bleeding (VARC-3 type ≥2)	44 (4.8%)	22 (5.1%)	22 (4.5%)	0.668
Severe heart failure	41 (4.4%)	22 (5.1%)	19 (3.9%)	0.372
New unplanned cardiac surgery	41 (4.5%)	36 (8.3%)	1 (0.2%)	**<0** **.** **001**
Severe delirium	41 (4.4%)	25 (5.1%)	16 (3.7%)	0.386
Stroke	27 (2.9%)	7 (1.6%)	17 (3.5%)	0.078
Major vascular complication	21 (2.3%)	1 (0.2%)	20 (4.1%)	**<0** **.** **001**
Cardiac arrest	6 (0.7%)	3 (0.7%)	3 (0.6%)	1.000
Major coronary artery obstruction	5 (0.5%)	4 (0.9%)	1 (0.2%)	0.192
Myocardial infarction	4 (0.4%)	3 (0.7%)	1 (0.2%)	0.346
Periprocedural death^a^	23 (2.5%)	11 (2.5%)	12 (2.4%)	1.000

VARC-3, Valve Academic Research Consortium-3.

^a^ Definitions are provided in the Supplementary Material.

Bold values indicate a significantly statistical difference.

^b^ Over *n* = 665 patients without previous atrial fibrillation.

^c^ Over *n* = 863 patients without previous cardiac implantable electronic device.

**Figure 2:**
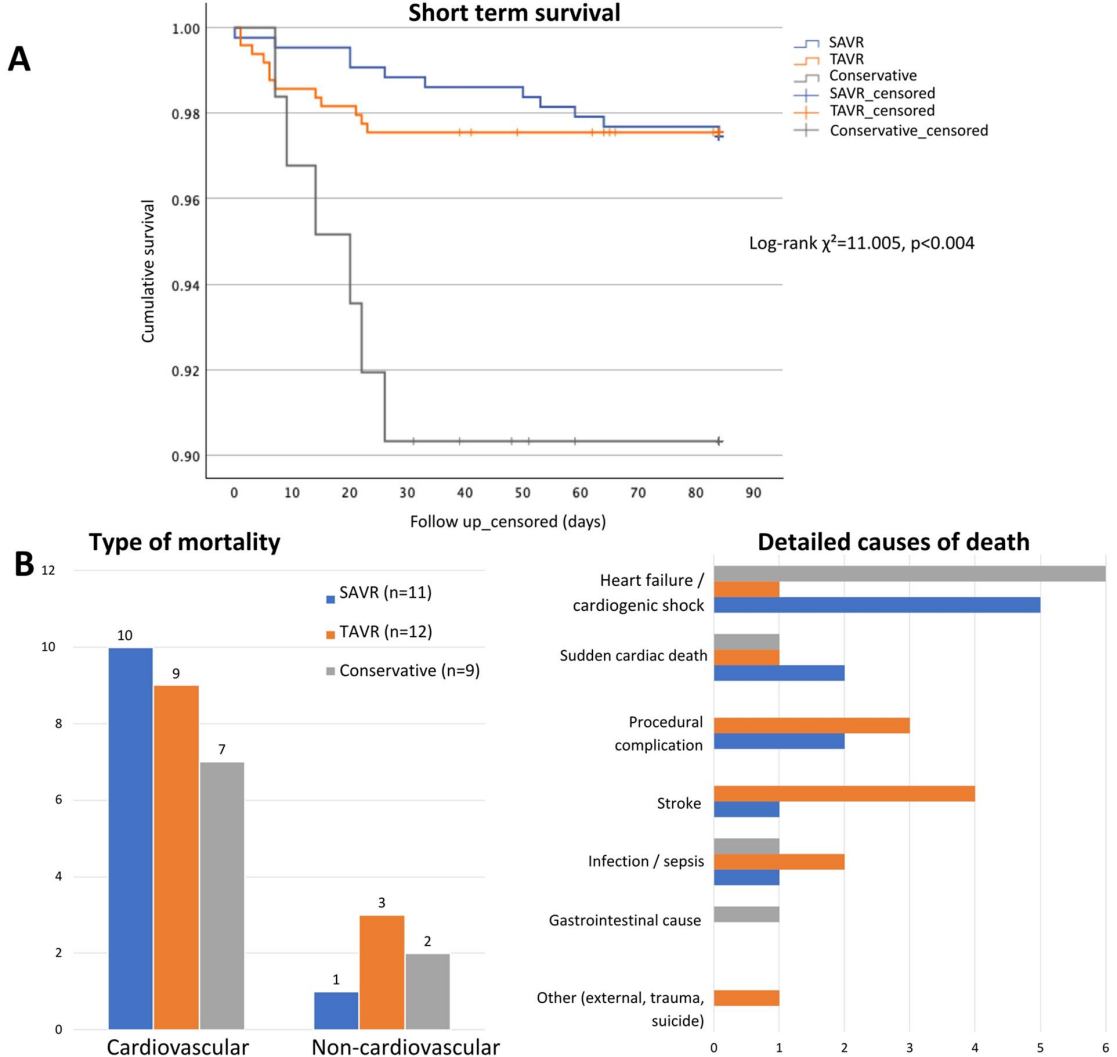
Early mortality and causes of death by treatment strategy. **(A)** Kaplan–Meier survival curves showing early mortality (up to 84 days) by treatment group. **(B)** Causes of death by treatment group, grouped as cardiovascular vs. non-cardiovascular (left) and detailed by specific cause (right). Abbreviations: as in Figure 1.

Figure 2A shows Kaplan–Meier survival curves across treatment strategies. Cumulative survival differed significantly between groups (log-rank test, *χ*^2^ = 11.005, *p* = 0.004), with the conservative group showing lower early survival (mean: 78 days, 95% CI: 72–83) compared with TAVR (mean: 82 days, 95% CI: 81–83) and SAVR (mean: 83 days, 95% CI: 82–84). Cardiovascular causes accounted for the majority of early deaths (79.3%), with distribution varying by treatment strategy. A detailed distribution of causes of death by treatment group is shown in Figure 2B.

## 4 Discussion

This study provides real-world evidence from a 5-year experience implementing an ICP supported by structured data management for patients with severe AS considered for valve replacement. Our model enabled comprehensive patient profiling and risk stratification, and facilitated proactive monitoring of clinical alerts and pre-procedural optimization. It also allowed for detailed evaluation of short-term health outcomes. Patients managed within the AS-ICP showed low preintervention mortality, high procedural success rates and low early mortality in both interventional arms, significantly lower than in the conservative group. Periprocedural complications differed by treatment strategy: permanent pacemaker implantation and major vascular complications were more frequent after TAVR, while surgical reintervention and new-onset atrial fibrillation were more common after SAVR. Notably, while nearly two thirds of patients in the interventional arms –particularly surgical patients– participated in the prehabilitation exercise program, postprocedural rehabilitation uptake was identified as a key area for improvement.

### 4.1 Integrated care pathways in aortic stenosis

Comprehensive evaluation of AS patients through multidisciplinary Heart Teams (18) and specialized management in valve centers is advocated in clinical practice guidelines (6, 7). Our findings further support a paradigm shift away from traditional procedure-centered models toward integrated disease management, addressing the entire patient journey –from diagnosis to intervention and rehabilitation– within a defined ICP. This model was successfully implemented in a tertiary hospital rather than a separated heart valve center, highlighting its feasibility in real-world practice. ICPs have shown to reduce care fragmentation and variability, promoting adherence to protocols and patient safety (8). In our cohort, this translated into remarkably low pre-intervention mortality (0.4%) compared to previous studies (11, 12).

Given the volume and variability of data generated throughout the ICP, structured data management was essential to ensure consistent documentation, longitudinal tracking, and reliable outcome analysis. The use of interoperable, ontology-based terminology allowed standardized data collection, while integrated rule-based clinical decision support tools facilitated automated calculations and inferences. This approach aligns with broader initiatives, such as the National Health Service (NHS) England's use of SNOMED CT to ensure uniform coding and data integration across institutions and national registries (19). Ultimately, high-quality structured datasets support participation in cross-system platforms such as the European Health Data Space, enabling large-scale research, public health monitoring, and predictive AI modeling (20).

### 4.2 Patient profile and AS phenotype

Our prospective cohort reflects the complexity of real-world clinical practice, including a high proportion of elderly and frail patients. Expected differences age, comorbidity burden, and surgical risk across treatment arms mirror those reported in large TAVR and SAVR registries (21, 22). However, few studies have included patients managed conservatively. The CURRENT-2 registry in Japan (2018–2020) (23) and the VALVENOR study in France (2016–2017) (24) both provided insights into this subgroup, showing, in line with our findings, that conservatively managed patients are generally older, frailer and more comorbid than interventional candidates.

Comprehensive frailty and functional status assessment is crucial to guide therapy in AS (25). In our cohort, we performed an extensive multiparametric geriatric evaluation in more than 80% of patients aged ≥75 years, demonstrating strong adherence to diagnostic protocols. This showed a stepwise gradient, with the worst scores in the conservative group, intermediate in TAVR and best in SAVR, reflecting an overall appropriate patient selection. However, patient preferences also influenced management decisions. For instance, 9 patients in the conservative group (14.5%) ultimately declined valve replacement (6 TAVR, 3 SAVR). Reported refusal rates in other registries vary from 7%–50% (24, 26, 27), likely due to heterogeneous definitions. In our study, only late refusals –after Heart Team presentation– were counted, which may explain the comparatively lower rate.

High-gradient AS was by far the predominant phenotype in our cohort. Low-gradient/low-flow AS was more frequent in the TAVR and conservative groups, consistent with previous studies linking these subtypes with higher comorbidity burden and greater frailty, as well as poorer clinical outcomes after valve replacement (27). Additionally, most patients in the interventional arms were in early cardiac damage stages (0–2), reflecting overall timely referral. In contrast, nearly 40% of those treated conservatively presented in stages 3–4, which are associated with worse survival rates after aortic valve replacement (28).

Median progression from moderate to severe AS was just over two years, in line with prior data (29). However, about one quarter of patients showed rapid progression, a pattern associated with worse outcomes (30) that may justify early intervention (6, 7). Strikingly, very severe AS was more common in our cohort (≈27%) than previously reported (15%–18%) (23, 31). This higher prevalence may reflect improved detection, but also a potential referral bias, or indirect effects of the COVID-19 pandemic. Despite prioritizing continued attention to severe AS, patient inclusion dropped significantly in 2020, likely due to reduced access to primary care, hospital referral, and delays in diagnostic testing and interventions (32, 33). Limited access to care may explain the higher-than-expected proportion of patients with very severe AS in 2020 and in the following years (34). Overall, these findings emphasize the need for early detection, timely referral and treatment, to limit advanced-stage presentations and to detect rapid progressors.

### 4.3 Diagnostic work-up and periprocedural optimization within the AS-ICP

Diagnostic work-up differed depending on the therapeutic approach, as expected. Of note, pyrophosphate scintigraphy to rule out TTTR cardiac amyloidosis was particularly common among TAVR patients, especially in 2019–2020, when the conduction of the AMY-TAVI study raised awareness of this condition (35). Also, patients treated conservatively underwent fewer invasive examinations, but almost 15% had stress testing to clarify symptoms and guide decisions (6, 7).

Periprocedural optimization included anemia and obesity management in more than 50% of eligible patients, while a prehabilitation exercise program was implemented in nearly two-thirds. Uptake was higher before SAVR, probably related to the younger, fitter profile of surgical candidates and longer pre-procedural waiting times. In contrast, despite growing evidence that structured CR programs improve functional recovery (36) and reduce hospitalizations and mortality (37), overall participation remained low—particularly after TAVR—as previously reported (38, 39). Lower engagement following TAVR may relate to greater comorbidity and frailty, transportation barriers, less routine referral, and lower perceived benefit in elderly patients (38–40). Moreover, participation dropped sharply during the COVID-19 pandemic, mirroring the disruption in CR services across Europe (38), with an encouraging but still modest rebound afterwards. These findings underscore the need to consider prehabilitation and CR as core components of valve care pathways, and to address barriers to patient participation. In this context, exploratory analyses suggested a potential association between prehabilitation and lower early mortality in our cohort, particularly in TAVR patients; however, the low event rate and lack of multivariable adjustment preclude definitive conclusions, and confirmation in larger studies is warranted.

### 4.4 Health outcomes of aortic valve replacement procedures

We observed very high technical and device success rates and low periprocedural mortality (≈2%). These results are consistent with large surgical series reporting 30-day or in-hospital mortality rates of 2%–3% for isolated SAVR and 3%–12% for combined interventions (22, 41). In our cohort, nearly half of SAVR patients underwent combined procedures, which may have influenced periprocedural outcomes and should be considered when interpreting comparisons with TAVR. In TAVR, despite the older age and higher-risk profile of patients, recent registries report 30-day or in-hospital mortality rates of 1%–3% (21, 22), matching our results. However, reported outcomes vary according to patient risk, type of prosthesis and vascular access.

Periprocedural complications increase length of stay, costs, and morbimortality (42). As expected, patterns differed between procedures. After TAVR, nearly 22% of patients required a permanent pacemaker, one of the most common complications due to the anatomical proximity of the aortic annulus to the His bundle (43). Risk factors include patient-related—older age, male sex, atrial fibrillation, previous right bundle branch block, or annular calcification—and procedural factors—self-expanding valves, greater oversizing or deep implantation (43, 44). The almost exclusive use of self-expanding valves at our center likely contributed to the relatively high incidence, which is consistent with randomized trials (45, 46) and large registries (47). Because pacemaker implantation after TAVR carries worse prognosis (43, 48), advances in valve designs, implantation techniques and pacing strategies are needed to optimize clinical outcomes.

Major vascular complications were also more frequent after TAVR (≈4%). Randomized trials reported rates of 2%–% after TAVR in low-risk (46, 49), and 6%–7% in intermediate-risk patients (45, 50), though using VARC-2 definitions (51). More recently, a multicenter cohort study reported a 7.4% incidence after transfemoral TAVR using VARC-3 criteria, mainly driven by vascular injury and closure device failure. The authors found a significant association with increased mortality at 30 days and one year, and identified several risk factors, including female sex (60% in our cohort), peripheral arterial disease, higher sheath-to-femoral ratio, dual antiplatelet therapy, and use of large plug-based closure devices (52).

In contrast, SAVR patients more often required reoperation for bleeding or tamponade, and developed new-onset postoperative atrial fibrillation (≈19%), although lower than the 30%–50% reported elsewhere (53, 54). Postoperative atrial fibrillation is associated with longer hospital stay and worse prognosis, and its reported incidence varies according to definition, rhythm monitoring system, patient profile and intervention (53). In our study, TAVR patients had continuous rhythm monitoring throughout their admission, whereas in SAVR patients this was generally limited to intensive care and not always extended to the ward. This may have led to missed short or asymptomatic episodes, partly explaining the comparatively lower incidence observed.

### 4.5 Strengths and limitations

To our knowledge, this is the first prospective study to provide a comprehensive description of baseline characteristics and short-term outcomes of patients with severe AS managed within a defined ICP. The single-center setting may limit generalizability, but the prospective inclusion of nearly 1,000 consecutive patients, with over 300 structured variables per patient compiled in an interoperable data platform, enhances internal validity and provides a reproducible framework. The design was broad in scope and not primarily focused on the procedural details of valve replacement; therefore, specific technical aspects are not reported. Nearly half of SAVR patients in our cohort underwent combined procedures, which may have influenced periprocedural outcomes and limits direct comparison with TAVR. In addition, the temporary substitution of in-person rehabilitation by telemonitored programs during the COVID-19 pandemic may have influenced patient participation and early outcomes. Moreover, some variables—such as detailed reasons for treatment refusal or participation in rehabilitation programs—were not consistently available, but data collection is being refined for future analyses. Finally, the relatively small number of conservatively managed patients limits conclusions specific to this subgroup, but reflects real-world Heart Team decision-making in the contemporary management of severe AS. Despite these limitations, the comprehensive clinical profiling across all treatment groups and the use of robust, standardized endpoints—facilitating consistency and comparability with other studies— provide valuable real-world insight into patient characteristics and short-term outcomes, while highlighting implementation challenges such as limited uptake of prehabilitation and cardiac rehabilitation.

## 5 Conclusions

Implementation of an integrated care pathway for aortic stenosis, supported by structured data management, in a tertiary hospital enabled comprehensive patient profiling, risk stratification, and early outcome monitoring. Both surgical and transcatheter interventions achieved high procedural success with low early mortality, while survival was poorer with conservative management. Engagement in prehabilitation was encouraging, while post-procedural rehabilitation remained underutilized, representing an important opportunity for improvement.

## Data Availability

The raw data supporting the conclusions of this article will be made available by the authors, without undue reservation.

## References

[1] Coffey S Roberts-Thomson R Brown A Carapetis J Chen M Enriquez-Sarano M Global epidemiology of valvular heart disease. Nat Rev Cardiol. (2021) 18:853–64. 10.1038/s41569-021-00570-z 34172950

[2] Iung B Delgado V Rosenhek R Price S Prendergast B Wendler O Contemporary presentation and management of valvular heart disease: the EUrobservational research programme valvular heart disease II survey. Circulation. (2019) 140:1156–69. 10.1161/CIRCULATIONAHA.119.041080 31510787

[3] Osnabrugge RLJ Mylotte D Head SJ Van Mieghem NM Nkomo VT LeReun CM Aortic stenosis in the elderly: disease prevalence and number of candidates for transcatheter aortic valve replacement: a meta-analysis and modeling study. J Am Coll Cardiol. (2013) 62:1002–12. 10.1016/j.jacc.2013.05.015 23727214

[4] Benfari G Essayagh B Michelena HI Ye Z Inojosa JM Ribichini FL Severe aortic stenosis: secular trends of incidence and outcomes. Eur Heart J. (2024) 45:1877–86. 10.1093/eurheartj/ehad887 38190428

[5] Généreux P Sharma RP Cubeddu RJ Aaron L Abdelfattah OM Koulogiannis KP The mortality burden of untreated aortic stenosis. J Am Coll Cardiol. (2023) 82:2101–9. 10.1016/j.jacc.2023.09.796 37877909

[6] Otto CM Nishimura RA Bonow RO Carabello BA Erwin JP Gentile F 2020 ACC/AHA guideline for the management of patients with valvular heart disease: a report of the American College of Cardiology/American Heart Association joint committee on clinical practice guidelines. Circulation. (2021) 143:E72–227. 10.1161/CIR.0000000000000923 33332150

[7] Praz F Borger MA Lanz J Marin-Cuartas M Abreu A Adamo M 2025 ESC/EACTS guidelines for the management of valvular heart disease: developed by the task force for the management of valvular heart disease of the European Society of Cardiology (ESC) and the European association for cardio-thoracic surgery (EACTS). Eur Heart J. (2025) 67:ezaf276. 10.1093/eurheartj/ehaf194

[8] Gartner JB Abasse KS Bergeron F Landa P Lemaire C Cote A. Definition and conceptualization of the patient-centered care pathway, a proposed integrative framework for consensus: a concept analysis and systematic review. BMC Health Serv Res. (2022) 22:558. 10.1186/s12913-022-07960-0 35473632 PMC9040248

[9] Veerle B Katrien D Bos P Remmen R Van Olmen J Wouters E. Development and operationalization of a data framework to assess quality of integrated diabetes care in the fragmented data landscape of Belgium. BMC Health Serv Res. (2022) 22:1257. 10.1186/S12913-022-08625-8 36253775 PMC9578257

[10] D’amore JD Mccrary LK Denson J Vitale CJ Tokachichu P Sittig DF Clinical data sharing improves quality measurement and patient safety. J Am Med Inform Assoc. (2021) 28:1534. 10.1093/jamia/ocab039 33712850 PMC8279795

[11] Albassam O Henning KA Qiu F Cram P Sheth TN Ko DT Increasing wait-time mortality for severe aortic stenosis: a population-level study of the transition in practice from surgical aortic valve replacement to transcatheter aortic valve replacement. Circ Cardiovasc Interv. (2020) 13:E009297. 10.1161/CIRCINTERVENTIONS.120.009297 33167700

[12] González Saldivar H Vicent Alaminos L Rodríguez-Pascual C de la Morena G Fernández-Golfín C Amorós C Prognosis of patients with severe aortic stenosis after the decision to perform an intervention. Rev Esp Cardiol (Engl Ed). (2019) 72:392–7. 10.1016/j.rec.2018.03.023 29997054

[13] Baumgartner H Hung J Bermejo J Chambers JB Edvardsen T Goldstein S Recommendations on the echocardiographic assessment of aortic valve stenosis: a focused update from the European association of cardiovascular imaging and the American society of echocardiography. Eur Heart J Cardiovasc Imaging. (2017) 18:254–75. 10.1093/ehjci/jew335 28363204

[14] Généreux P Pibarot P Redfors B Mack MJ Makkar RR Jaber WA Staging classification of aortic stenosis based on the extent of cardiac damage. Eur Heart J. (2017) 38:3351–8. 10.1093/eurheartj/ehx381 29020232 PMC5837727

[15] Généreux P Piazza N Alu MC Nazif T Hahn RT Pibarot P Valve academic research consortium 3: updated endpoint definitions for aortic valve clinical research. Eur Heart J. (2021) 42:1825–57. 10.1093/eurheartj/ehaa799 33871579

[16] Larrañaga-Moreira JM Rodriguez-Serrano AI Domínguez F Lalario A Zorio E Barriales-Villa R. Impact of SARS-CoV-2 infection in patients with cardiac amyloidosis: results of a multicentre registry. Med Clin (Barc). (2023) 161:476–82. 10.1016/j.medcli.2023.06.025 37684159

[17] Gimeno JR Olivotto I Rodríguez AI Ho CY Fernández A Quiroga A Impact of SARS-cov-2 infection in patients with hypertrophic cardiomyopathy: results of an international multicentre registry. ESC Heart Fail. (2022) 9:2189–98. 10.1002/ehf2.13964 36255281 PMC9288745

[18] Costa C Teles RC Brito J Neves JP Gabriel HM Abecassis M Advantages of a prospective multidisciplinary approach in transcatheter aortic valve implantation: eight years of experience. Rev Port Cardiol. (2017) 36:809–18. 10.1016/j.repc.2016.11.015 29153618

[19] SNOMED CT—NHS England Digital. (n.d.). Available online at: https://digital.nhs.uk/services/terminology-and-classifications/snomed-ct (Accessed August 5, 2025).

[20] European Health Data Space Regulation (EHDS)—European Commission. (n.d.). Available online at: https://health.ec.europa.eu/ehealth-digital-health-and-care/european-health-data-space-regulation-ehds_en (Accessed August 5, 2025).

[21] Nguyen V Willner N Eltchaninoff H Burwash IG Michel M Durand E Trends in aortic valve replacement for aortic stenosis: a French nationwide study. Eur Heart J. (2022) 43:666–79. 10.1093/eurheartj/ehab773 34849714

[22] Lundahl C Kragholm K Tayal B Karasoy D Andersen NH Strange JE Temporal trends in patient characteristics and outcomes of transcatheter aortic valve implantation and surgical aortic valve replacement: a nationwide study. Am J Cardiol. (2024) 211:299–306. 10.1016/j.amjcard.2023.11.024 37984636

[23] Takeji Y Taniguchi T Morimoto T Shirai S Kitai T Tabata H Rationale, design, and baseline characteristics of the CURRENT AS registry-2. Circ J. (2022) 86:1769–76. 10.1253/circj.CJ-21-1062 35444112

[24] Coisne A Montaigne D Aghezzaf S Ninni S Lemesle G Sudre A Clinical outcomes according to aortic stenosis management: insights from real-world practice. J Am Heart Assoc. (2024) 13:36657. 10.1161/JAHA.124.036657 PMC11681392 39548024

[25] Niebauer J Bäck C Bischoff-Ferrari HA Dehbi H-M Szekely A Völler H Preinterventional frailty assessment in patients scheduled for cardiac surgery or transcatheter aortic valve implantation: a consensus statement of the European association for cardio-thoracic surgery (EACTS) and the European association of preventive cardiology (EAPC) of the European Society of Cardiology (ESC). Eur J Prev Cardiol. (2024) 31:146–81. 10.1093/eurjpc/zwad304 37804173

[26] Ishii M Taniguchi T Morimoto T Ogawa H Masunaga N Abe M Reasons for choosing conservative management in symptomatic patients with severe aortic stenosis—observations from the CURRENT AS registry. Circ J. (2019) 83:1944–53. 10.1253/circj.CJ-19-0247 31316039

[27] Taniguchi T Morimoto T Takeji Y Shirai S Ando K Tabata H Low-Gradient severe aortic stenosis: insights from the CURRENT AS registry-2. JACC Cardiovasc Interv. (2025) 18:471–87. 10.1016/j.jcin.2024.09.044 39708011

[28] Abdelfattah OM Jacquemyn X Sá MP Jneid H Sultan I Cohen DJ Cardiac damage staging predicts outcomes in aortic valve stenosis after aortic valve replacement: meta-analysis. JACC Adv. (2024) 3:100959. 10.1016/j.jacadv.2024.100959 38939639 PMC11198616

[29] Willner N Prosperi-Porta G Lau L Nam Fu AY Boczar K Poulin A Aortic stenosis progression: a systematic review and meta-analysis. JACC Cardiovasc Imaging. (2023) 16:314–28. 10.1016/j.jcmg.2022.10.009 36648053

[30] Benfari G Nistri S Marin F Cerrito LF Maritan L Tafciu E Excess mortality associated with progression rate in asymptomatic aortic valve stenosis. J Am Soc Echocardiogr. (2021) 34:237–44. 10.1016/j.echo.2020.11.015 33253813

[31] Angelillis M Costa G Primerano C Giannini C Adamo M Chizzola G Outcomes of patients with very severe aortic stenosis treated with transcatheter aortic valve implantation. Am J Cardiol. (2023) 205:241–8. 10.1016/j.amjcard.2023.07.148 37611417

[32] Williams MC Shaw L Hirschfeld CB Maurovich-Horvat P Nørgaard BL Pontone G Impact of COVID-19 on the imaging diagnosis of cardiac disease in Europe. Open Heart. (2021) 8:e001681. 10.1136/openhrt-2021-001681 34353958 PMC8349647

[33] Romaguera R Ojeda S Cruz-González I Moreno R Guisado Rasco A Gutiérrez-Barrios A Spanish Cardiac catheterization and coronary intervention registry. 30th official report of the interventional cardiology association of the Spanish society of cardiology (1990–2020) in the year of the COVID-19 pandemic. Rev Esp Cardiol (Engl Ed). (2021) 74:1095–105. 10.1016/j.rec.2021.10.008 34782287 PMC8552543

[34] Ooms JF Hokken TW Adrichem R Gunes D de Ronde-Tillmans M Kardys I Changing haemodynamic status of patients referred for transcatheter aortic valve intervention during the COVID-19 pandemic. Neth Heart J. (2023) 31:399–405. 10.1007/s12471-023-01795-y 37498468 PMC10516812

[35] Bastos-Fernandez M Lopez-Otero D Lopez-Pais J Pubul-Nuñez V Neiro-Rey C Lado-Baleato O Echocardiographic phenotype in severe aortic stenosis with and without transthyretin cardiac amyloidosis: the AMY-TAVI study. Eur Heart J Cardiovasc Imaging. (2025) 26:261–72. 10.1093/ehjci/jeae263 39437308

[36] Hosseinpour A Azami P Hosseinpour H Attar A Koushkie Jahromi M. Efficacy of exercise training-based cardiac rehabilitation programmes after transcatheter aortic valve implantation: a systematic review and meta-analysis. Int J Cardiol Cardiovasc Risk Prev. (2024) 20:200238. 10.1016/j.ijcrp.2024.200238 38322761 PMC10844670

[37] Patel DK Duncan MS Shah AS Lindman BR Greevy RA Savage PD Association of cardiac rehabilitation with decreased hospitalization and mortality risk after cardiac valve surgery. JAMA Cardiol. (2019) 4:1250–9. 10.1001/jamacardio.2019.4032 31642866 PMC6813589

[38] Sukul D Albright J Thompson MP Villablanca P Keteyian SJ Yaser J Predictors and variation in cardiac rehabilitation participation after transcatheter aortic valve replacement. JACC Adv. (2023) 2:100581. 10.1016/j.jacadv.2023.100581 38938330 PMC11198261

[39] Guduguntla V Yaser JM Keteyian SJ Pagani FD Likosky DS Sukul D Variation in cardiac rehabilitation participation during aortic valve replacement episodes of care. Circ Cardiovasc Qual Outcomes. (2022) 15:E009175. 10.1161/CIRCOUTCOMES.122.009175 35559710 PMC10068673

[40] González-Salvado V Peña-Gil C Lado-Baleato Ó Cadarso-Suárez C Prada-Ramallal G Prescott E Offering, participation and adherence to cardiac rehabilitation programmes in the elderly: a European comparison based on the EU-CaRE multicentre observational study. Eur J Prev Cardiol. (2021) 28:558–68. 10.1093/eurjpc/zwaa104 33558875

[41] Kundi H Popma JJ Granada JF Leon MB Kodesh A Ascione G Outcomes in older patients undergoing surgical aortic valve replacement with concomitant procedures. J Am Coll Cardiol. (2025) 86:280–3. 10.1016/j.jacc.2025.05.021 40701672

[42] Harvey JE Ryan M Gunnarsson C Chikermane S Baron SJ. Incremental cost of complications after TAVR and SAVR in contemporary clinical practice. Am Heart J. (2025) 290:278–87. 10.1016/j.ahj.2025.07.001 40659198

[43] Nuche J Ellenbogen KA Mittal S Windecker S Benavent C Philippon F Conduction disturbances after transcatheter aortic valve replacement: an update on epidemiology, preventive strategies, and management. JACC Cardiovasc Interv. (2024) 17:2575–95. 10.1016/j.jcin.2024.07.032 39603774

[44] Hosseini Mohammadi NS Tavakoli K Taebi M Zafari A Riahi M Molaei MM Comparative prognostic value of risk factors for predicting pacemaker implantation after transcatheter aortic valve replacement: a systematic review and network meta-analysis. Am J Cardiol. (2025) 250:79–89. 10.1016/j.amjcard.2025.05.009 40348046

[45] Reardon MJ Van Mieghem NM Popma JJ Kleiman NS Søndergaard L Mumtaz M Surgical or transcatheter aortic-valve replacement in intermediate-risk patients. N Engl J Med. (2017) 376:1321–31. 10.1056/NEJMoa1700456 28304219

[46] Popma JJ Deeb GM Yakubov SJ Mumtaz M Gada H O’Hair D Transcatheter aortic-valve replacement with a self-expanding valve in low-risk patients. N Engl J Med. (2019) 380:1706–15. 10.1056/NEJMoa1816885 30883053

[47] Pagnesi M Kim WK Baggio S Scotti A Barbanti M De Marco F Incidence, predictors, and prognostic impact of new permanent pacemaker implantation after TAVR with self-expanding valves. JACC Cardiovasc Interv. (2023) 16:2004–17. 10.1016/j.jcin.2023.05.020 37480891

[48] Badertscher P Stortecky S Serban T Knecht S Heg D Tueller D Long-Term outcomes of patients requiring pacemaker implantation after transcatheter aortic valve replacement: the SwissTAVI registry. JACC Cardiovasc Interv. (2025) 18:1163–71. 10.1016/j.jcin.2025.03.028 40368460

[49] Mack MJ Leon MB Thourani VH Makkar R Kodali SK Russo M Transcatheter aortic-valve replacement with a balloon-expandable valve in low-risk patients. N Engl J Med. (2019) 380:1695–705. 10.1056/NEJMoa1814052 30883058

[50] Leon MB Smith CR Mack MJ Makkar RR Svensson LG Kodali SK Transcatheter or surgical aortic-valve replacement in intermediate-risk patients. N Engl J Med. (2016) 374:1609–20. 10.1056/NEJMoa1514616 27040324

[51] Kappetein AP Head SJ Généreux P Piazza N van Mieghem NM Blackstone EH Updated standardized endpoint definitions for transcatheter aortic valve implantation: the valve academic research consortium-2 consensus document. Eur Heart J. (2012) 33:2371–76. 10.1093/eurheartj/ehs255 23026477

[52] Cepas-Guillén P Avvedimento M Ternacle J Urena M Alperi A Cheema AN Vascular complications in patients undergoing transcatheter aortic valve replacement with contemporary devices. Can J Cardiol. (2025) 41:1490–502. 10.1016/j.cjca.2025.03.034 40204013

[53] Gaudino M Di Franco A Rong LQ Piccini J Mack M. Postoperative atrial fibrillation: from mechanisms to treatment. Eur Heart J. (2023) 44:1020–39. 10.1093/eurheartj/ehad019 36721960 PMC10226752

[54] Ryan T Grindal A Jinah R Um KJ Vadakken ME Pandey A New-Onset atrial fibrillation after transcatheter aortic valve replacement: a systematic review and meta-analysis. JACC Cardiovasc Interv. (2022) 15:603–13. 10.1016/j.jcin.2022.01.018 35331452

