# Supplementary material for: Implementation of an integrated care pathway for severe aortic stenosis: a prospective cohort study of clinical profiles, safety, and outcomes
Source: Front Cardiovasc Med. 2026 Jan 16;12:1745204. doi: 10.3389/fcvm.2025.1745204 (PMC12856929; doi:10.3389/fcvm.2025.1745204)
Supplement: Supplementary file 1 [file Datasheet1.pdf]

Supplementary Material

I. Supplementary figures and tables

Supplementary Figure 1. Reappraised decisions and change of therapeutic plan

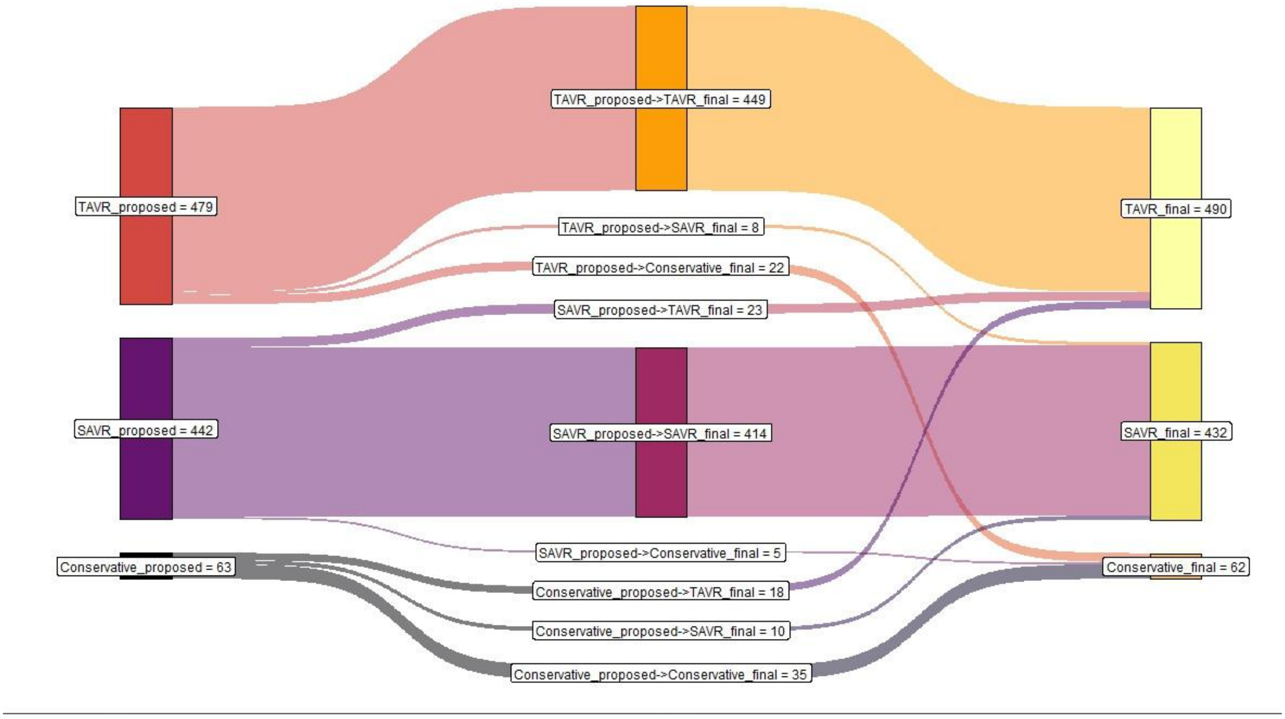

Supplementary table 1. Diagnostic studies performed within the AS-ICP by treatment group

	Total (n=984)	SAVR (n=432)	TAVR (n=490)	Conservative (n=62)	p-value
**Diagnostic tests**					
Coronary angiography	932 (94.3%)	426 (99.4%)	460 (93.8%)	46 (74.2%)	**<0.001**
Cardiac CT angiography	640 (65.0%)	118 (27.3%)	489 (99.8%)	34 (54.8%)	**<0.001**
TEE	183 (18.6%)	108 (25.0%)	63 (12.9%)	12 (19.4%)	**<0.001**
Non-contrast chest CT	182 (18.5%)	103 (23.8%)	69 (14.1%)	10 (16.1%)	**<0.001**
Pyrophosphate scintigraphy	104 (10.6%)	10 (2.3%)	91 (18.6%)	3 (4.8%)	**<0.001**
Cardiac stress testing*	96 (9.8%)	52 (12.0%)	35 (7.1%)	9 (14.5%)	**<0.001**
Dobutamine stress test	54 (5.5%)	40 (9.3%)	11 (2.2%)	3 (4.5%)	
Exercise stress test	42 (4.3%)	12 (2.8%)	24 (4.9%)	6 (9.7%)	
SAT doppler ultrasound	65 (6.6%)	45 (10.4%)	18 (3.7%)	2 (3.2%)	**<0.001**
CRM	12 (1.2%)	7 (1.6%)	4 (0.8%)	1 (1.6%)	0.518
Right heart catheterization	11 (1.1%)	3 (0.7%)	5 (1.0%)	3 (4.8%)	**0.014**

Abbreviations: CT, computerized tomography; TEE, transesophageal echocardiogram; SAT, supraaortic trunks; CMR, cardiovascular magnetic resonance. *Stress test, the results of which determined the indication for intervention or assessment by the Heart Team.

Supplementary figure 2. Diagnostic studies performed within the AS-ICP by year

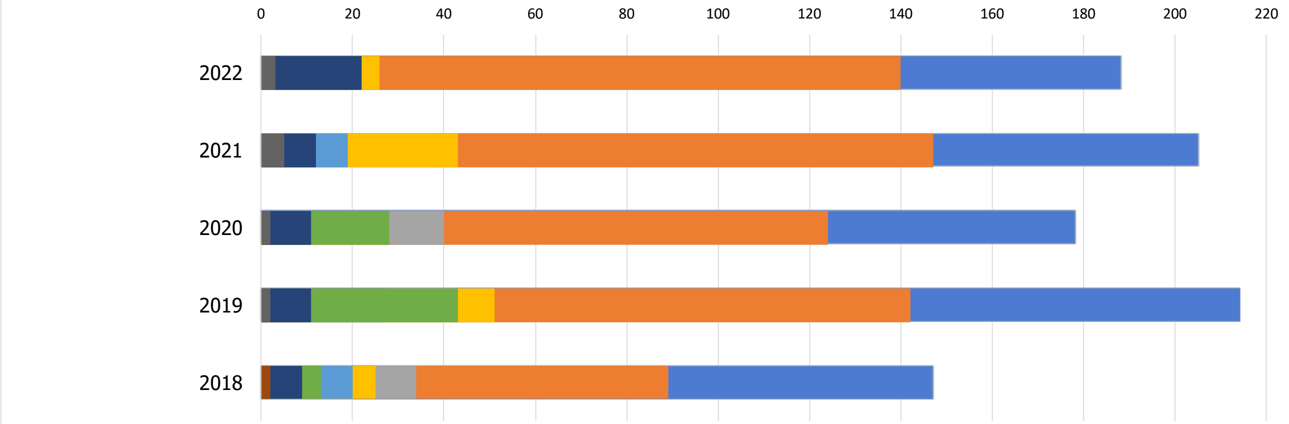

	2018	2019	2020	2021	2022
CMR	0	2	2	5	3
Right catheterization	2	1	1	4	3
SAT doppler US	9	11	11	12	22
Pyrophosphate scintigraphy	13	43	28	8	12
Stress test	20	27	10	19	20
TEE	25	51	28	43	26
Non-contrast chest CT	34	45	40	38	25
Cardiac angioCT	89	142	124	147	140
Coronary angiography	147	214	178	205	188

CMR, Right catheterization, SAT doppler US, Pyrophosphate scintigraphy, Stress test, TEE, Non-contrast chest CT, Cardiac angioCT, Coronary angiography

Supplementary figure 3. Proportion of patients undergoing aortic valve replacement who received in each supportive intervention within the AS-ICP by year

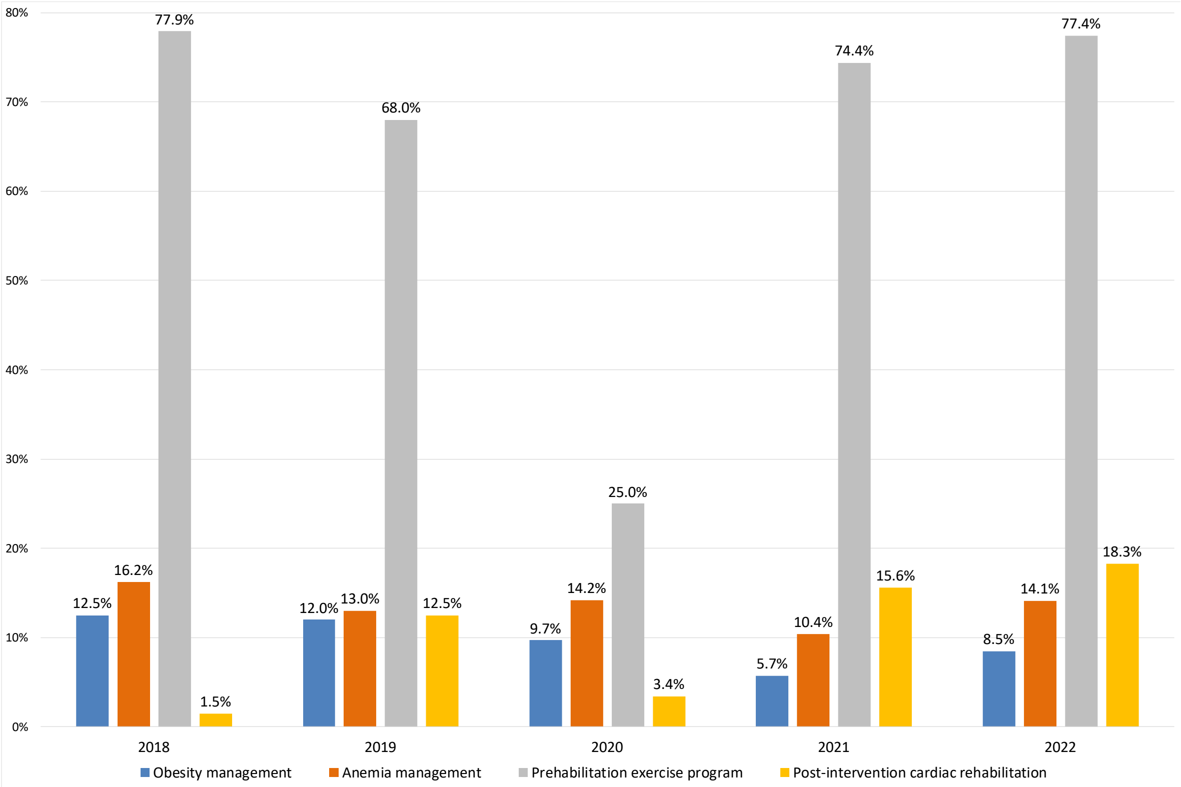

Supplementary Figure 4. Characteristics of aortic valve replacement procedures

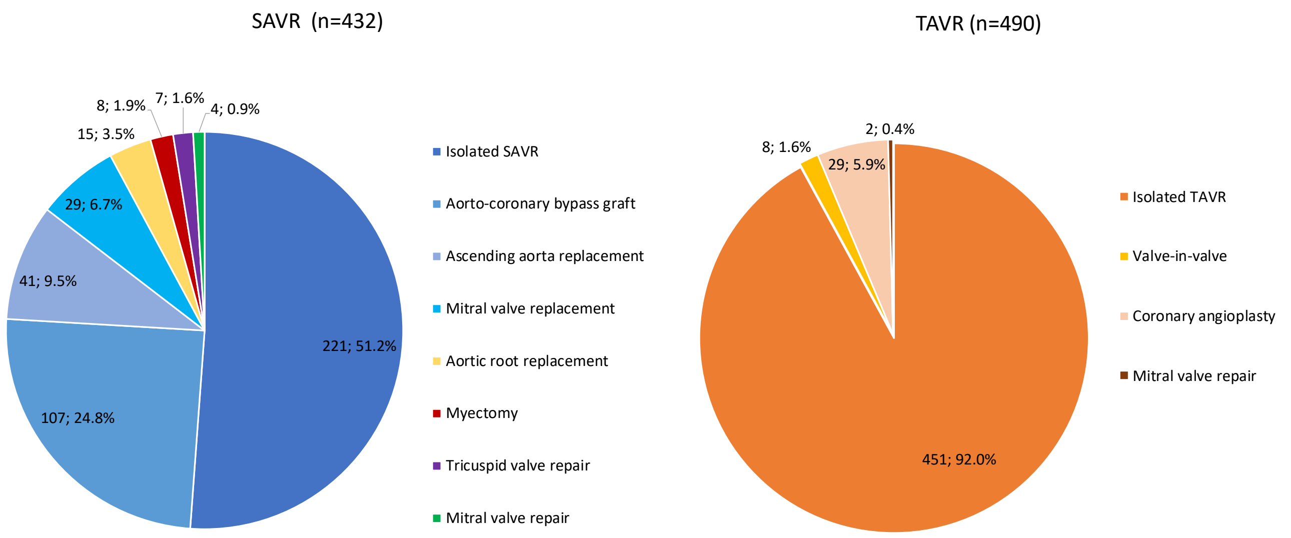

Supplementary Figure 5. Length of hospital stay by type of intervention and patient origin

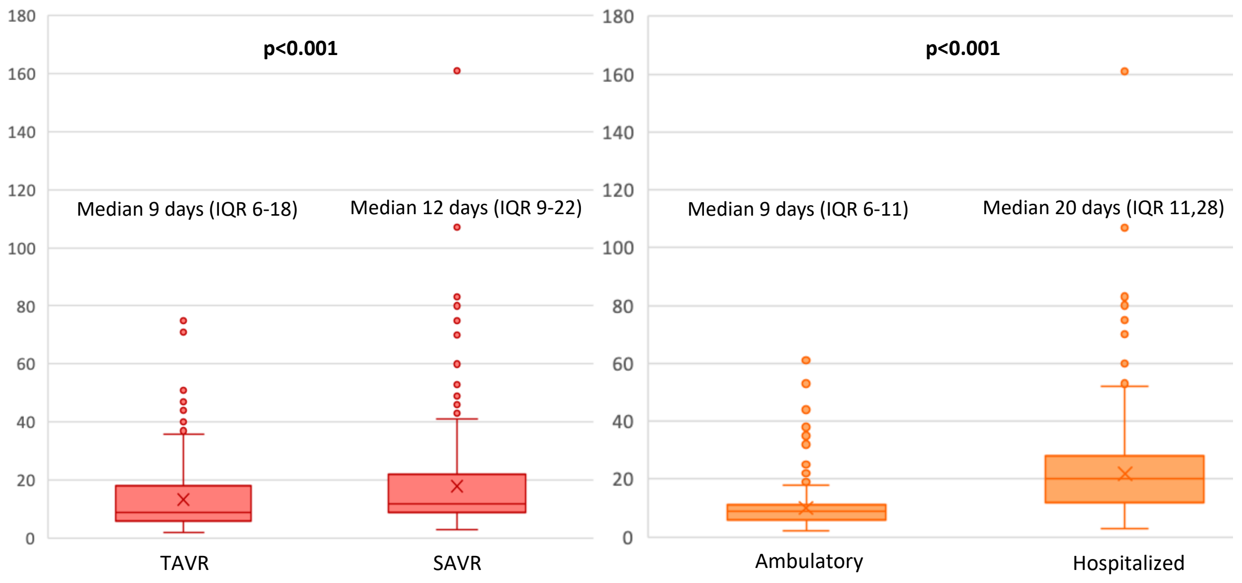

II. Other supplementary material

2.1 Multidimensional geriatric assessment protocol

Assessment tool	Score and interpretation	References
**Functional independence & social support**		
Barthel index	90–100: Independent<br>60–90: Mild dependence<br>40–55: Moderate dependence<br>20–35: Severe dependence<br><20: Total dependence	Mahoney FI, Barthel DW. Functional evaluation: the Barthel Index. Md State Med J. 1965;14:61–5.
Gijón scale	≤7: Good social situation<br>8–9: Intermediate situation<br>≥10: Severe social deterioration	Miralles R, Sabartés O, Ferrer M, Esperanza A, Llorach I, García-Palleiro P, Cervera AM. Development and validation of an instrument to predict probability of home discharge from a geriatric convalescence unit in Spain. J Am Geriatr Soc. 2003;51(2):252–7.
**Cognitive & nutritional status**		
Mini Nutritional Assessment Test Short Form (MNA-SF)	12–14: Normal nutritional status<br>8–11: Risk of malnutrition<br>0–7: Malnutrition	Rubenstein LZ, Harker JO, Salva A, Guigoz Y, Vellas B. Screening for undernutrition in geriatric practice: developing the short-form mini-nutritional assessment (MNA-SF). J Gerontol A Biol Sci Med Sci. 2001;56(6):M366–72.
Mini-Mental Test (adapted by Lobo et al.)	25–30: Normal<br>19–24: Mild cognitive impairment<br>14–18: Moderate impairment<br><14: Severe impairment	Lobo A, Saz P, Marcos G, Díaz JL, de la Cámara C, Ventura T, et al. Revalidación y normalización del Mini-Examen Cognoscitivo (versión española del Mini-Mental State Examination). Med Clin (Barc). 1999;112(20):767–74.
**Frailty & physical performance**		
FRAIL scale	0: Robust<br>1–2: Pre-frail<br>3–5: Frail	Morley JE, Malmstrom TK, Miller DK. A simple frailty questionnaire (FRAIL) predicts outcomes in middle aged African Americans. J Nutr Health Aging. 2012;16(7):601–8.
Essential Frailty Tooltest	0: Robust<br><3: Not frail<br>≥3: Frail	Afilalo J, Lauck S, Kim DH, Lefèvre T, Piazza N, Lachapelle K, et al. Frailty in older adults undergoing aortic valve replacement: the FRAILTY-AVR study. J Am Coll Cardiol. 2017;70(6):689–700.
Gait speed (m/s)	<0.8 m/s: Frail<br>≥0.8 m/s: Not frail	Studenski S, Perera S, Patel K, Rosano C, Faulkner K, Inzitari M, et al. Gait speed and survival in older adults. JAMA. 2011;305(1):50–8.
Short Physical Performance Battery	0–4: Severe limitation<br>4–6: Moderate limitation<br>7–9: Mild limitation<br>10–12: Minimal limitation	Guralnik JM, Ferrucci L, Simonsick EM, Salive ME, Wallace RB. Lower-extremity function in persons over the age of 70 years as a predictor of subsequent disability. N Engl J Med. 1995;332(9):556–61.
6-min walking test (m)	<300 m: Frail<br>≥300 m: Not frail	ATS Committee on Proficiency Standards for Clinical Pulmonary Function Laboratories. ATS statement:

6-min walking test (%)	No established cut-off point for the studied population	guidelines for the six-minute walk test. Am J Respir Crit Care Med. 2002;166(1):111–7.<br>Kanamori N, Takeji Y, Taniguchi T, Morimoto T, Shirai S, Ando K, et al. Prognostic value of the 6-minute walk test in patients with severe aortic stenosis. Circ J. 2025. Epub ahead of print. doi:10.1253/circj.CJ-25-0056.

2.2 Periprocedural complications and adverse events

Complications are reported according to **VARC-3** updated criteria whenever possible (Généreux P, Piazza N, Alu MC, Nazif T, Hahn RT, Pibarot P, et al. Valve Academic Research Consortium 3: updated endpoint definitions for aortic valve clinical research. Eur Heart J. 2021;42:1825-1857).

**Procedural success**	
**Technical success (at exit from procedure room)**	1) Freedom from mortality<br>2) Successful access, device delivery and delivery system retrieval<br>3) Correct positioning of a single prosthetic valve in the proper anatomical location<br>4) No surgery or intervention related to the device or to a major vascular or access-related, or cardiac structural complication.
**Device success (≤30 days)**	§ Fulfills al technical success criteria, plus: intended valve performance (mean gradient <20mmHg, peak velocity <3 m/s, Doppler velocity index ≥0.25, and less than moderate aortic regurgitation) at 30 days.
**Periprocedural complications (at 30 days or during index hospitalization)**	
**Permanent pacemaker implantation**	Permanent pacemaker implantation after aortic valve intervention due to conduction disturbances. Rate calculation excludes patients with previous cardiac implantable electronic devices.
**New-onset atrial fibrillation**	New-onset atrial fibrillation after aortic valve intervention which lasts sufficiently long to be recorded on a 12-lead ECG or at least 30 seconds on a rhythm strip. Rate calculation excludes patients with previous atrial fibrillation.
**Major bleeding**	Overt bleeding VARC-3 type ≥2:<br>***Type 2***<br>• Requiring transfusion of 2–4 units of whole blood/red blood cells (BARC 3a)<br>• Associated with a hemoglobin drop of >3 g/dL (>1.86 mmol/L) but <5 g/d (<3.1 mmol/L) (BARC 3a)<br>***Type 3***<br>• Critical organ overt bleeding associated with hemodynamic compromise/ tamponade and necessitating intervention, or intramuscular with compartment syndrome (BARC 3b/c)<br>• Causing hypovolemic shock or severe hypotension or requiring vasopressors or surgery (BARC 3b)<br>• Requiring reoperation, surgical exploration, or re-intervention for the purpose of controlling bleeding (BARC 3b/4)<br>• Post-thoracotomy chest tube output ≥2 L within a 24-h period (BARC 4)<br>• Requiring transfusion of ≥5 units of whole blood/red blood cells (BARC 3a)<br>• Associated with a haemoglobin drop ≥5 g/dL (≥3.1 mmol/L) (BARC 3b)

	**Type 4**<br>• Leading to death Probable (BARC 5a) or definite (BARC 5b)
**Severe heart failure**	Includes any of the following:<br>1) Signs and symptoms consistent with pulmonary edema or cardiogenic shock<br>2) Hemodynamic compromise requiring high-dose inotropes or vasopressors for >24 hours post-procedure or need for mechanical circulatory support, with low cardiac output syndrome with signs of end-organ dysfunction<br>3) Prolonged mechanical ventilation or ICU stay due to heart failure symptoms, or initiation or escalation of heart failure therapies beyond routine postoperative care
**New unplanned cardiac surgery**	Any cardiac surgical intervention related to the index procedure, including interventions due to valve-related complications (i.e., paravalvular leak, prosthesis dysfunction, or infective endocarditis) as well as procedures for non-valve-related issues such as postoperative bleeding or cardiac tamponade.
**Severe delirium**	Transient non-focal neurological signs or symptoms without evidence of infarction on neuroimaging or pathology, or with no imaging performed, corresponding to a CAM-ICU scale of 6-7.
**Stroke**	• Ischaemic stroke: Acute onset of focal neurological signs or symptoms conforming to a focal or multifocal vascular territory within the brain, spinal cord, or retina and fulfilling one of the following criteria:<br>- Signs or symptoms lasting >_24 h or until death, with pathology or neuroimaging evidence of CNS infarction, or absence of other apparent causes<br>- Symptoms lasting <24 h, with pathology or neuroimaging confirmation of central nervous system infarction in the corresponding vascular territory<br>• Haemorrhagic stroke: Acute onset of neurological signs or symptoms due to non-traumatic intracranial bleeding (intracerebral or subarachnoid haemorrhage)<br>• Stroke, not otherwise specified: Acute onset of neurological signs or symptoms persisting ≥24 h or until death but without sufficient neuroimaging or pathology evidence to be classified
**Major vascular complication**	Includes any of the following:<br>• Aortic dissection or aortic rupture<br>• Vascular injury or compartment syndrome resulting in death, VARC type ≥2 bleeding, limb or visceral ischaemia, or irreversible neurologic impairment<br>• Distal embolization (non-cerebral) from a vascular source resulting in death, amputation, limb or visceral ischaemia, or irreversible end-organ damage<br>• Unplanned endovascular or surgical intervention resulting in death, VARC type ≥2 bleeding, limb or visceral ischaemia, or irreversible neurologic impairment<br>• Closure device failure resulting in death, VARC type ≥2 bleeding, limb or visceral ischaemia, or irreversible neurologic impairment
**Cardiac arrest**	Sudden cessation of cardiac mechanical activity confirmed by the absence of signs of circulation.
**Major coronary artery obstruction**	New partial or complete obstruction of a coronary ostium or an epicardial coronary artery during or after the procedure, caused either by the valve prosthesis, native leaflets, embolized material, external device compression, or coronary artery instrumentation, with objective evidence of ischaemia or symptoms resulting in death, haemodynamic compromise, myocardial infarction, or unplanned surgical or percutaneous intervention.

**Myocardial infarction**	Acute myocardial injury demonstrated by elevated cardiac biomarkers (cardiac troponin) with a rise and/or fall of values, together with clinical evidence of acute myocardial ischemia.
**Periprocedural death**	Death within 30 days of the index procedure or later if still hospitalized.

2.3 STROBE checklist for cohort studies

	Item No	Recommendation	Page No
**Title and abstract**	1	(*a*) Indicate the study's design with a commonly used term in the title or the abstract	1
		(*b*) Provide in the abstract an informative and balanced summary of what was done and what was found	4
**Introduction**			
Background/rationale	2	Explain the scientific background and rationale for the investigation being reported	5,6
Objectives	3	State specific objectives, including any prespecified hypotheses	6
**Methods**			
Study design	4	Present key elements of study design early in the paper	6
Setting	5	Describe the setting, locations, and relevant dates, including periods of recruitment, exposure, follow-up, and data collection	6
Participants	6	(*a*) Give the eligibility criteria, and the sources and methods of selection of participants. Describe methods of follow-up	6-8
		(*b*) For matched studies, give matching criteria and number of exposed and unexposed	Not applicable
Variables	7	Clearly define all outcomes, exposures, predictors, potential confounders, and effect modifiers. Give diagnostic criteria, if applicable	6-8
Data sources/ measurement	8	For each variable of interest, give sources of data and details of methods of assessment (measurement). Describe comparability of assessment methods if there is more than one group	7,8
Bias	9	Describe any efforts to address potential sources of bias	20
Study size	10	Explain how the study size was arrived at	6
Quantitative variables	11	Explain how quantitative variables were handled in the analyses. If applicable, describe which groupings were chosen and why	8
Statistical methods	12	(*a*) Describe all statistical methods, including those used to control for confounding	8
		(*b*) Describe any methods used to examine subgroups and interactions	9-15
		(*c*) Explain how missing data were addressed	14
		(*d*) If applicable, explain how loss to follow-up was addressed	Not applicable

		(*e*) Describe any sensitivity analyses	Not applicable
**Results**			
Participants	13	(a) Report numbers of individuals at each stage of study—eg numbers potentially eligible, examined for eligibility, confirmed eligible, included in the study, completing follow-up, and analysed<br>(b) Give reasons for non-participation at each stage<br>(c) Consider use of a flow diagram	8-9<br>9<br>Reported in the text
Descriptive data	14	(a) Give characteristics of study participants (eg demographic, clinical, social) and information on exposures and potential confounders<br>(b) Indicate number of participants with missing data for each variable of interest<br>(c) Summarise follow-up time (eg, average and total amount)	10-13<br>14<br>8
Outcome data	15	Report numbers of outcome events or summary measures over time	15-16

Main results	16	(*a*) Give unadjusted estimates and, if applicable, confounder-adjusted estimates and their precision (eg, 95% confidence interval). Make clear which confounders were adjusted for and why they were included<br>(*b*) Report category boundaries when continuous variables were categorized<br>(*c*) If relevant, consider translating estimates of relative risk into absolute risk for a meaningful time period	15-16<br>Not applicable<br>Not applicable
Other analyses	17	Report other analyses done—eg analyses of subgroups and interactions, and sensitivity analyses	13-18
**Discussion**			
Key results	18	Summarise key results with reference to study objectives	16
Limitations	19	Discuss limitations of the study, taking into account sources of potential bias or imprecision. Discuss both direction and magnitude of any potential bias	20
Interpretation	20	Give a cautious overall interpretation of results considering objectives, limitations, multiplicity of analyses, results from similar studies, and other relevant evidence	20
Generalisability	21	Discuss the generalisability (external validity) of the study results	20
**Other information**			
Funding	22	Give the source of funding and the role of the funders for the present study and, if applicable, for the original study on which the present article is based	2
